# A Genomics-Guided Multimodal Contrastive Learning Framework for Clinically Significant Prostate Cancer Risk Stratification with Missing Clinical Data

**DOI:** 10.3390/cancers18121952

**Published:** 2026-06-16

**Authors:** Abdullah, Muhammad Shahid, Muhammad Ateeb Ather, Zulaikha Fatima, Carlos Guzmán Sánchez Mejorada, Miguel Jesús Torres Ruiz, Rolando Quintero Téllez, Miguel Félix Mata-Rivera, Roberto Zagal-Flores

**Affiliations:** 1 Centro de Investigación en Computación (CIC), Instituto Politécnico Nacional (IPN), Mexico City 07738, Mexico; abdullah2025@cic.ipn.mx (A.); cmejorada@cic.ipn.mx (C.G.S.M.); mtorres@cic.ipn.mx (M.J.T.R.); 2 Department of Computer Sciences, Bahria University, Lahore 54600, Pakistan; mshahid.bulc@bahria.edu.pk; 3 Faculty of Allied Health Sciences, Superior University, Lahore 54000, Pakistan; su91-bmitm-f23-248@superior.edu.pk; 4 Interdisciplinary Professional Unit in Engineering and Advanced Technologies (UPIITA), Instituto Politécnico Nacional (IPN), Mexico City 07340, Mexico; mmatar@ipn.mx; 5 Higher School of Computing (ESCOM), Instituto Politécnico Nacional (IPN), Mexico City 07738, Mexico; rzagalf@ipn.mx

**Keywords:** prostate cancer, multimodal learning, genomics-anchored deep learning, radio-genomics, histopathology data, contrastive learning, risk stratification, clinical decision support, calibration, interpretable AI

## Abstract

Background: Heterogeneous data integration remains a major challenge in intelligent information systems, particularly under missing-modality and cross-domain conditions. Existing multimodal fusion approaches often rely on complete datasets and weak alignment mechanisms, limiting their robustness and practical applicability. Objectives: This study aims to develop and evaluate a genomics-guided multimodal representation learning framework that enables robust heterogeneous data fusion, reliable cross-modal correspondence, and accurate prediction under incomplete-data conditions. Methods: We propose a multimodal learning architecture that models genomics as the primary biological anchor and learns conditional projections to imaging modalities, including multiparametric MRI and whole-slide histopathology (WSI). The framework formulates multimodal fusion as a genomics-guided contrastive learning problem, incorporates domain-specific optimization constraints, and learns a latent shared-state representation to support inference without requiring fully paired datasets. Evaluation was conducted using public datasets, including TCGA-PRAD and TCIA, across low-risk versus higher-risk/clinically significant prostate cancer (csPCa) discrimination, Gleason-based risk stratification, and clinically significant outcome prediction tasks under realistic multimodal and missing-modality scenarios. Results: In the adequately powered Genomics+WSI cohort (*n* = 486), the framework achieved an AUROC of 0.985 ± 0.005 for low-risk versus higher-risk/csPCa discrimination (*p* < 0.001). Exploratory analysis in a small, matched Genomics+MRI cohort (*n* = 28) yielded an AUROC of 0.980 ± 0.006 for the same endpoint; these findings are reported descriptively with bootstrap confidence intervals due to limited sample size. Because the negative reference group consisted of low-risk prostate cancer cases rather than cancer-free controls, results are interpreted as within-cancer risk discrimination rather than de novo cancer detection. The framework achieved weighted accuracy up to 92.1%, Cohen’s κ up to 0.86, and reduced critical decision errors by 58%. Calibration remained strong (ECE 0.021–0.024), and decision-curve analysis indicated improved utility with reduced unnecessary invasive workups in retrospective modeling. Robustness analysis demonstrated AUROC degradation below 0.04 under domain shifts. Single-modality inference using genomics alone maintained AUROC > 0.90. Interpretability analysis revealed feature attributions aligned with domain-relevant genomic markers. Conclusions: The proposed framework provides a scalable and generalizable solution for heterogeneous multimodal data fusion, supporting reliable prediction, robustness to missing modalities, and applicability to complex information systems beyond the studied domain.

## Simple Summary

This research aims to improve how different types of medical data, including genetic information, MRI scans, and tissue images, can be combined to support accurate and reliable clinically significant prostate cancer risk stratification in patients with confirmed prostate cancer, especially when some data are missing or collected from different sources. The authors develop a flexible artificial intelligence framework that uses genetic information as the main guide for learning relationships between multiple data types without requiring complete patient records. The proposed system is designed to improve low-risk versus higher-risk/csPCa discrimination, reduce critical risk-stratification errors, and remain effective in resource-limited settings where only one type of data may be available. These findings may help researchers build more robust and scalable multimodal learning systems for healthcare and other complex information domains, while improving interpretability and reliability in real-world decision-support applications.

## 1. Introduction

Prostate cancer (PCa) is still one of the most widespread cancers in men all over the world and the main cause of morbidity and mortality rates due to cancer [1]. In spite of significant screening, imaging, and molecular profiling, the challenge of risk stratification and early detection of the disease of clinical significance remains a significant challenge [2]. The modern-day practice of clinicians incorporates a blend of prostate-specific antigen (PSA) test, multiparametric magnetic resonance imaging (mpMRI), and histopathologic evaluation after biopsy [3]. Although each modality offers a source of valuable information, none of them is enough to fully define the biological heterogeneity and clinical complexity of prostate cancer.

Artificial intelligence (AI) has become a powerful tool for supporting prostate cancer diagnosis, prognosis, and risk stratification across imaging, histopathology, and molecular data. Deep-learning models have achieved expert-level performance in mpMRI-based lesion detection and whole-slide image (WSI)-based Gleason grading, while genomics-based models have improved molecular subtyping and outcome prediction [4]. Recent interpretable multi-dataset learning approaches and intelligent hybrid reinforcement learning frameworks have demonstrated the value of integrating heterogeneous clinical and biomedical data for robust healthcare prediction and decision-making under uncertainty [5,6]. However, most current prostate cancer AI strategies remain modality-centric, treating imaging, histopathology, and genomics as separate prediction tasks or combining them through late-stage feature concatenation. This type of strategy ignores the causal biology of cancer, in which genomic perturbations are the drivers of subsequent phenotypic phenomena that can be observed in radiologic and histopathologic data [7].

The more recent multimodal learning models seek to cope with these shortcomings through the combination of heterogeneous sources of data. However, the majority of multimodal models of prostate cancer customs presuppose the complete access of all modalities, a weakly coupled fusion mechanism, or are not biology-based [8]. Practically, the triple-modality data (genomics, MRI, and histopathology) are not commonly provided on the same patient, and the practical clinical decisions should be made with incomplete and uncertain evidence [9,10]. In addition, traditional fusion techniques run a risk of learning spurious associations due to modality-specific noise, scanner change, or institutional bias, and restricting generalization and clinical credibility [11].

Genomic and epigenomic changes before and during prostate cancer are an underlying force of the future progress of the cancer, which determines radiologic features and tissue morphology [12,13]. Imaging and histopathology are modeled as phenotypic projections of the underlying molecular state, consistent with the biological hierarchy [14]. Although such a hierarchy is clearly established, genomics is rarely coded into existing AI systems as a key organizing principle to multimodal representation learning. Consequently, the existing models have a hard time separating molecular heterogeneity and spatial and morphological heterogeneity, especially in missing-modality conditions [15].

In this study, we propose a genomics-anchored multimodal learning framework for prostate cancer that unifies radiologic and histopathologic representations through a shared molecular backbone. Rather than treating multimodal fusion as post hoc feature aggregation, we formulate it as biologically guided representation alignment, where patient-specific genomic embeddings serve as a stable latent anchor and imaging modalities, including mpMRI and WSI, are modeled as conditional phenotypic expressions of the same disease state. The framework is evaluated in two clinically realistic settings, Genomics+MRI and Genomics+WSI, without assuming simultaneous availability of all modalities. Cross-modal contrastive learning aligns each imaging modality to the genomic latent space, while modality dropout enables robust genomics-only inference when imaging is unavailable. Clinical priors, including PI-RADS, Gleason grading, and NCCN risk-stratification, are integrated as optimization constraints to support medically consistent predictions. Evaluation on public prostate cancer cohorts demonstrates improved low-risk versus higher-risk/csPCa discrimination and risk stratification performance over unimodal and conventional fusion baselines, alongside enhanced domain generalization and biologically interpretable cross-modal representations.

Unlike existing multimodal prostate cancer models that treat genomics and imaging as parallel inputs fused at the feature or decision level, our framework is explicitly designed around a genomics-anchored latent space derived from TCGA-PRAD molecular profiles. In this architecture, RNA-seq, CNV, and DNA methylation embeddings form a biologically grounded reference manifold that guides cross-modal alignment of matched mpMRI and H&E whole-slide representations. Rather than simple concatenation or independent task heads, the model employs contrastive cross-modal alignment coupled with clinically constrained multi-task optimization to jointly learn low-risk versus higher-risk/csPCa discrimination, Gleason-based risk stratification, and clinically significant prostate cancer risk prediction within a unified objective. Importantly, robustness is not treated as a post hoc evaluation step; instead, scanner-level, institution-level, and acquisition-protocol domain shifts, missing-modality scenarios, and calibration-aware training are incorporated into the design and validation pipeline. This framework systematically models genotype–radiophenotype–histophenotype interactions through a genomics-centered multimodal learning strategy, addressing an important gap in current prostate cancer AI approaches. By explicitly incorporating generalization and clinical consistency constraints, it provides a promising transferable multimodal AI paradigm, pending prospective external validation.

Together, this contribution gains prostate cancer AI a step forward through the presentation of a multimodal learning paradigm that is biologically based, clinically sensitive, and deployment-ready. The main contributions of this work are summarized as follows:

1. We introduce a biology-first multimodal learning framework that treats genomics as the causal anchor of prostate cancer and models MRI and histopathology as conditional phenotypic projections, departing fundamentally from conventional modality-centric fusion strategies.
2. We propose a genomics-centered contrastive learning mechanism that independently aligns genomic embeddings with MRI and WSI representations, enabling effective multimodal learning without requiring fully matched genomics–MRI–WSI data.
3. The framework supports Genomics+MRI, Genomics+WSI, and genomics-only inference through modality dropout and hierarchical fusion, ensuring robustness under real-world incomplete data scenarios.
4. We integrate PI-RADS consistency, Gleason grading coherence, and NCCN risk stratification directly into the training objective via differentiable constraints, enhancing clinical plausibility and trustworthiness of predictions.
5. We conduct extensive experiments, ablation studies, generalization testing, and explainability analyses on public datasets, demonstrating improved performance, robustness, and interpretability compared to state-of-the-art unimodal and multimodal baselines.

By combining biological grounding, modality-agnostic representations, and clinical constraint awareness, this work defines a coherent and generalizable class of multimodal medical AI systems for clinically significant prostate cancer risk assessment and stratification. The remainder of this paper is organized as follows. It first reviews related work in prostate cancer AI and multimodal learning. Next, it presents the proposed genomics-anchored architecture and learning strategy. This is followed by a description of the experimental setup, datasets, and evaluation protocols. The subsequent part reports quantitative results, ablation studies, and explainability analyses. The discussion then highlights clinical implications, limitations, and future research directions, and the paper concludes with final remarks.

## 2. Related Work

Recent progress in prostate cancer (PCa) studies underlines the growing importance of artificial intelligence in patient education, imaging, molecular profiling, and multimodal integration. Alasker et al. [16] assessed large language models in the area of patient-facing PCa education, showing a high level of accuracy and readability and highlighting that the latter tools can be used as a supplement, but not as a decision-making tool. In addition to language models, methodological innovation also continues in the field of imaging-based diagnostics, such as the PROS-TD-AI protocol by Ramos et al. [17], combining time-dependent diffusion MRI metrics to overcome some of the limitations of classical mpMRI. Tapper et al. [18] have provided a review of AI use in molecular imaging of prostate cancer with a focus on PSMA-based PET/CT and MRI. The article reviews radiomics and deep-learning tools in diagnostic and prognostic tasks, but it is still limited to the field of imaging without incorporating genomic or histopathologic information. Such developments coincide with more general architectural developments surveyed by Pu et al. [19], who have noted that transformer and hybrid CNN-transformer models are strong at encoding long-range dependencies with smaller data sets, which is a challenge that can be found everywhere in medical AI.

In-depth reviews also highlight the potential and deficiencies of the existing PCa AI systems. Significant methodological heterogeneity and lack of external validation of deep-learning studies were found by Talyshinskii et al. [20], which supports the importance of standardized testing and strong testing of generalization. The availability of data is a severe bottleneck; Adams et al. [21] offer expert-marked datasets of prostate MRI, and Kratzer et al. [22] give epidemiological evidence of the increasing advanced-stage incidences and long-standing racial inequalities, which define the necessity to find ways to improve early detection methods. Bakht and Beltran [23] examined the intricate regulation and heterogeneity of PSMA expression on the biological level and emphasized why single-modality imaging methods might become inadequate in the correct stratification.

Multimodal learning has become an influential paradigm in order to overcome these difficulties. Esteva et al. [24] showed that the combination of histopathology and clinical data could be more effective in the long-term outcome prediction as compared to conventional NCCN risk stratification. In a similarly handling study, Zhang et al. [25] demonstrated that incorporating the knowledge of clinical guidelines with multimodal large language models can lead to the enhancement of PI-RADs scoring without further annotations, which demonstrates the importance of embedding domain knowledge in pipelines of imaging. Innovation is also facilitated by public resources like FastMRI Prostate [26] that offer raw k-space data to conduct advanced reconstructions and AI studies. To achieve a single heterogeneous modality, Simionescu [27] suggested Medformer, which is a foundation model that can be adapted to imaging tasks and anatomies, expounding the possibility of generalist multimodal architectures.

Instead, direct evidence of the benefits of multimodal performance is given by Jahanandish et al. [28], who demonstrated that the integration of MRI and transrectal ultrasound is more effective than unimodal models and radiologists in terms of lesion localization and specificity. In a systematic review study on machine learning and deep-learning approaches to prostate cancer diagnosis on medical images, Olabanjo et al. [29] pursued PRISMA guidelines on four large databases. The review has determined that the most popular modality is MRI, and the most popular methodology is transferring learning, but the limitations of datasets, the variability of the methodology, and the necessity to have a standardized evaluation and multimodal integration have been mentioned. The syntheses of evidence, proposed by Leenen et al. [30] to support risk-adjusted PCa screening with biomarkers and MRI, are not a new idea since the broader advantages of context-aware multimodal AI systems integrating heterogeneous biomedical and sensor-driven information for complex healthcare management have also been demonstrated by Fatima et al. [31].

Cross-modal fusion strategies are still in development. The SwinCross proved to be an essential building block of cross-modal attention used by Li et al. [32] to combine PET and CT, providing a universal blueprint of multimodal feature exchange that can be adapted to MRI-genomic or MRI-WSI fusion. The molecular taxonomy of TCGA, as described by Abeshouse et al. [33], demonstrated a high level of genomic heterogeneity in primary PCa, which is a solid biological rationale supporting the use of genomics in modeling. Degadwala et al. [34] showed that, in tasks that are pathology-specific, hybrid CNN-transformer models can attain high levels of accuracy in automated Gleason grading, which again confirms that WSI is a predictive modality of high quality. Lastly, Shahzad et al. [35] summarized the recent AI developments in PCa diagnosis and early detection, finding that further developments require strong multimodal integration and clinically oriented validation.

Together, this research makes multimodal learning with genomics underpinning, combining MRI and WSI, a logical and logical extension of prostate cancer AI. Contrary to previous literature, which relies on the single mode or loosely coupled fusion, the current research contributes to the literature by integrating genomic, radiologic, and histopathologic signals into a strictly validated, generalization-conscious model, directly relying on the limitations detected in the literature.

## 3. Research Methodology

**Figure 1:**
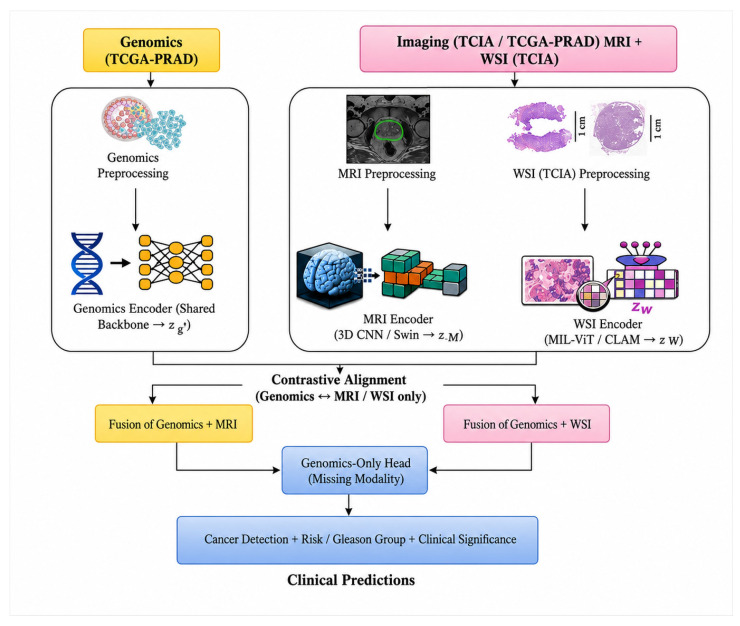
Data-flow Pipeline Diagram.

This study proposes a genomics-based multimodal learning model that combines heterogeneous molecular and imaging data to stratify clinically significant prostate cancer (csPCa) risk, as illustrated in Figure 1 and evaluated in two complementary settings: Genomics+MRI and Genomics+Histopathology. The framework employs a shared genomic encoder as a biologically grounded latent anchor, while modality-specific encoders process multiparametric MRI and whole-slide histopathology images as conditional phenotypic projections aligned through contrastive and task-supervised objectives. A unified multi-task head jointly optimizes discrimination between low-risk and higher-risk/csPCa cases, risk stratification, and clinically significant disease prediction, with clinically informed constraints incorporated to enforce guideline-consistent outputs. Training is performed using patient-level stratified cross-validation with modality dropout to support inference under missing-data conditions. All experiments were conducted on NVIDIA A100 GPUs, with training times of approximately 30–35 h per setting, and optimized inference pipelines enabling scalable deployment with sub-second latency for genomics-only prediction. This design promotes biological coherence, computational efficiency, and robustness across heterogeneous clinical data scenarios.

Importantly, the proposed design extends beyond dataset construction or modality pairing. The core contribution lies in the formulation of a genomics-anchored latent space that structurally constrains multimodal alignment and multi-task optimization. Rather than treating multimodal data as interchangeable inputs, the framework defines a biologically grounded representation manifold that governs cross-modal contrastive learning, task coupling, and modality dropout dynamics. This architectural formulation is modality-agnostic and transferable, enabling application to other multi-omics and imaging settings without altering the underlying optimization principles. Thus, the contribution resides in the representation learning strategy and constrained multi-task objective, not merely in dataset integration.

### 3.1. Data Acquisition

The proposed multimodal prostate cancer framework was compiled based on high-quality publicly available datasets and grouped based on complementary multimodal environments. The genomic data used were the TCGAPRAD cohort [36], which consists of RNA-sequencing expression, copy-number variation, and DNA methylation data with clinical annotations such as Gleason score, tumor stage, and clinically important disease status. Genomics, in the framework, plays the role of the common biological foundation, providing patient-specific molecular embeddings, which provide a platform for the learning of multimodal representations.

The imaging data have been obtained in genomically matched cohorts. Multiparametric prostate MRI data were obtained in the TCIA TCGA-PRAD imaging data repository [37] and consisted of T2-weighted, diffusion-weighted, and apparent diffusion coefficient data, thus providing anatomical and functional complementary data of the tumor. At the same time, images of whole amorphous histopathology with the use of staining on hematoxylin and eosin (H&E) were acquired from TCGA−PRAD and provided cellular and tissue-level morphological characterization. Combining these modalities allows modeling genotype–phenotype comprehensively on molecular, radiologic, and histopathologic scales.

For the purpose of this study, clinically significant prostate cancer (csPCa) was defined as pathologically confirmed Gleason Grade Group ≥ 2 (i.e., Gleason score ≥ 3 + 4 = 7), consistent with the consensus definition used in current EAU and NCCN guidelines and widely adopted in computational pathology studies. Progression risk was operationalized using NCCN risk-stratification categories (low, intermediate, high) derived from the combination of Gleason score, pre-treatment PSA level, and clinical stage (T-category). For biopsy decision modeling in the decision-curve analysis (Section 4.3), the clinically relevant threshold range was set at 5–30% probability of csPCa, consistent with the threshold range used in PI-RADS-guided biopsy referral guidelines. All labels were extracted from TCGA-PRAD clinical annotation files and independently verified against linked pathology reports where available.

### 3.2. Data Preprocessing

Genomic data have been preprocessed to give biologically consistent and steady representations. The low-expressing genes were filtered out, high-noise genes were filtered out, and missing values were imputed using biologically informed strategies. Counts of RNA-sequencing underwent log-transformation and standardization, either using z-score or the min-max scaling, to address the effects of batch and variability of measurements. Conditional on requirement, dimensionality reduction was performed using variance thresholds, filtering based on correlations, or the use of prior biological information known prostate cancer driver genes, thus improving the quality of downstream embedding or reducing dimensionality, as shown in Figure 1.

Multiparametric MRI scans were resampled to homogenous voxel spacing, intensity normalized per-sequence as T2w, DWI, and ADC, and cropped or padded to limit to the prostate. They were augmented using rotation, flipping, and elastic deformation, and optional ROI extraction was used to highlight lesions. Whole-slide images (WSIs) were tiled into 512 × 512 patches and then the background filtered, Macenko color normalized, and tumor-enriched. There was cohesiveness in the clinical labels used across modalities with a standardized encoding scheme to achieve consistent multimodal results.

The shared genomic encoder created patient-specific embeddings based on expression, CNV, and methylation data, which acted as a set of consistent biological benchmarks. Representations based on MRI were modeled with the 3D convolutional neural networks or transformer architectures Swin, and those of whole-slide images were produced with the multiple-instance learning (MIL)-based encoders as MIL-ViT or CLAM. The resultant imaging embeddings were then aligned to the genomic embeddings to rank patterns that are meaningful biologically.

### 3.3. Data Fusion Strategy

The framework was tested in two complementary multimodal designs, namely Genomics+MRI and Genomics+WSI as shown in Table 1. One common genomic encoder was used in both settings, thus eliminating the need to do forced triple-mode fusion and maintain methodological rigor and clinical plausibility.

Primary encoder used in all reported experiments: 3D Swin Transformer for Genomics + MRI setting; MIL-ViT for Genomics + WSI setting. The 3D CNN (MRI) and CLAM (WSI) alternatives were evaluated only in ablation studies to assess architecture-specific contributions, whereas all primary experiments employed the 3D Swin Transformer for MRI and MIL-ViT for WSI as the standard configuration.

Embedding level fusion was performed to enable interaction of genomic and imaging stage and prediction level fusion was maintained when interpretability was mandatory. Contrastive learning aligned genomics with each imaging modality, improving cross-modal consistency and robustness. Modality dropout promoted accurate inferences where data were not observed (Genomics+MRI, Genomics+WSI, genomicsonly) and therefore reflected the typical clinical practices of the real world and allowed generalization across modalities using robust methods.

### 3.4. Proposed Model Architecture

**Figure 2:**
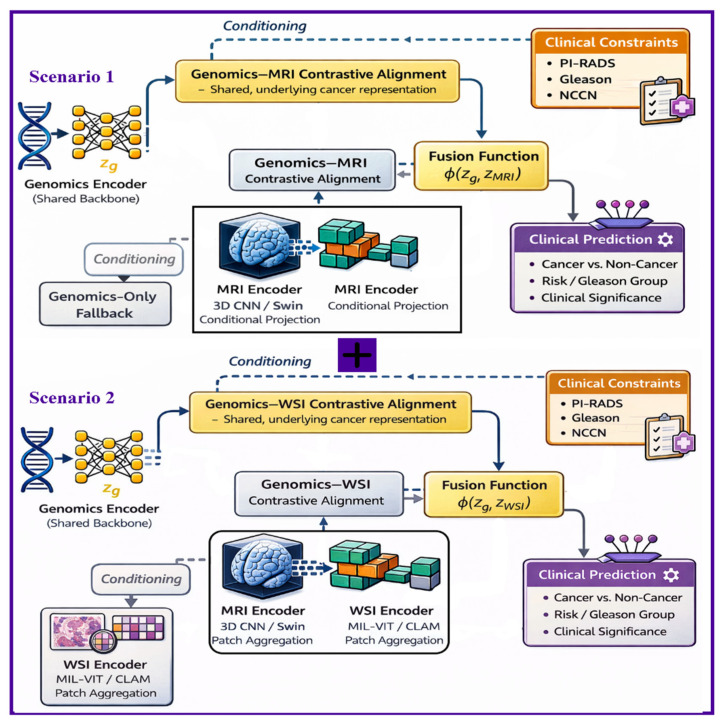
Proposed Multimodal Learning Architecture.

The proposed architecture addresses one of the main shortcomings of the previous prostate cancer AI systems by replacing late-stage multimodal fusion with a biologically justified representation alignment, as shown in Figure 2. Genomics, MRI, and histopathology are placed in this paradigm as the causal and downstream phenotypic expressions of genomic changes, PTEN, TP53, and SPOP, respectively. This system learns modality-agnostic latent cancer representation, thus allowing the system to perform robust inferences even in the absence of modalities, and optimize the system through clinical guidelines and biological plausibility to minimize spurious correlations. The observation of the preeminence of the genomics anchor is empirically supported by the experiments of genomic ablation, which consider non-genomic effects as noise.

Let g denote the latent genomic embedding and $x_{m}$ denote modality-specific observations. The learning objective assumes a causal hierarchy, as shown in Equation (1):

(1) $$p\left(x_{m}|g\right)\gg \;p\left(g|x_{m}\right),\;\;m\;\;\in \;\left\{MRI,\;WSI\right\}$$

and seeks a modality-invariant cancer representation z, such as shown in Equation (2):

(2) $$z\;=\;f\left(g\right),\;z\;⟂\;m$$

Throughout this section, $z_{g}$ denotes the genomic embedding, $z_{m}$ modality-specific embeddings, and $z_{fused}$ the final representation used for prediction.

1. The proposed architecture comprises a shared genomic encoder, modality-specific phenotypic encoders for MRI and whole-slide histopathology (WSI), and a contrastive alignment mechanism operating in a shared latent space. Rather than enforcing simultaneous fusion of all modalities, the framework is designed around two complementary multimodal settings, such as Genomics+MRI and Genomics+WSI, both anchored by the same genomic backbone. This design enables cross-setting regularization while avoiding assumptions of fully matched triple-modality data.
2. To respect real-world data availability, training proceeds in two stages. Stage A (modality-specific pretraining) independently pretrains phenotypic encoders on large unimodal datasets: ProstateX, PI-CAI, and related TCIA collections; WSI and genomics: TCGA-PRAD. Stage B (contrastive alignment and fine-tuning) applies genomics–imaging contrastive learning only to patients with matched data. For Genomics+MRI, where matched samples are limited, alignment relies on few-shot fine-tuning and transfer learning from the MRI-only pretrained encoder. No direct MRI–WSI alignment is performed.
3. During Stage B, optimization is set-wise. For each mini-batch, a setting S ∈{Genomics+MRI,Genomics+WSI} is sampled based on data availability, and only the modalities present in that setting are loaded. Latent representations are computed as $z_{g}=E_{g}\left(G\right)$, $z_{m}^{S}=E_{m}^{\left(S\right)}\left(X_{m}\right)$, where $E_{g}$ is shared across settings and $E_{m}^{\left(S\right)}$ is modality-specific. Losses are computed only for the active setting, ensuring setting-specific contrastive alignment and preventing artificial cross-modality coupling.

#### 3.4.1. Genomics-Centered Backbone Encoder

The genomic backbone integrates RNA-seq, CNV, and DNA methylation into a unified, patient-specific molecular embedding that captures tumor state independent of imaging. Cross-omics attention models interactions across molecular signals, while dimensionality-adaptive encoding suppresses noise and batch effects. The shared latent space encodes aggressiveness, subtype, and progression risk, enabling interpretable gene-level analysis and enforcing biological consistency across all multimodal settings. Given multi-omics inputs $G\;=\;\left\{G^{r},\;G^{c},\;G^{m}\right\}$, the genomic embedding is computed, as shown in Equation (3):

(3) $$z_{g}=\;Attn\left(E_{r}\left(G^{r}\right),\;E_{c}\left(G^{c}\right),\;E_{m}\left(G^{m}\right)\right)$$

where Attn(.) denotes cross-omics attention.

#### 3.4.2. Phenotypic Encoders as Conditional Projections

MRI and histopathology are modeled as conditional phenotypic projections of shared cancer biology rather than independent modalities. A 3D MRI encoder captures spatial–functional tumor patterns conditioned on genomics, suppressing scanner artifacts. A MIL-based WSI encoder aggregates patch morphology into slide-level features aligned to molecular phenotypes. Modalities are never directly fused, avoiding spurious cross-imaging correlations. Phenotypic embeddings are learned as conditional projections, as shown in Equation (4):

(4) $$z_{m}\;=\;E_{m}(X_{m}|z_{g})$$

ensuring that imaging representations are explicitly modulated by molecular context.

#### 3.4.3. Cross-Modal Contrastive Alignment

The core innovation is a genomics-anchored contrastive alignment that independently aligns MRI–genomics and WSI–genomics embeddings, rather than enforcing cross-imaging similarity. This yields a modality-agnostic latent cancer space where biologically similar tumors converge regardless of evidence source, regularizes imaging encoders against modality-specific noise, and uncovers phenotype–genotype associations without explicit supervision. Crucially, training does not require triple-modality data: each iteration activates a single multimodal setting, computes contrastive loss for the available modality, and updates both the shared genomic encoder and the corresponding phenotypic encoder, with the genomic backbone receiving gradients across settings, as shown in Equation (5):

(5) $$L_{con}^{\left(S\right)}=\;-\log\left(\frac{\exp\left(\frac{sim\left(z_{g},\;z_{m}^{\left(S\right)}\right)}{\tau }\right)}{\sum ⱼ\exp\left(\frac{sim\left(z_{g},\;z_{m_{j}}^{\left(S\right)}\right)}{\tau }\right)}\right)$$

And define the total contrastive objective, as shown in Equation (6):

(6) $$L_{con}=\;E_{S}\left[\;L_{con}^{S}\right]$$

At each training iteration, contrastive alignment is applied only to the imaging modality available in the active multimodal setting, avoiding any artificial alignment between MRI and histopathology. The hierarchy is conceptual, not mandatory. In practice, fusion is conditional on modality availability, as shown in Equation (7):

(7) $$z_{fused}\;=\;\left\{\begin{array}{c} \;\phi (z_{g},\;z_{MRI})\;\;\;\;\;\;if\;Genomics+MRI\\ \phi (z_{g},\;z_{WSI})\;\;\;\;\;\;if\;Genomics+WSI\\ z_{g}\;\;\;\;\;\;\;\;\;\;\;\;\;\;\;\;\;if\;Genomics\;only \end{array}\right.$$

The hierarchical fusion strategy is adaptive and collapses gracefully in the absence of downstream modalities, ensuring consistent inference under partial evidence.

#### 3.4.4. Progressive and Hierarchical Fusion Strategy

**Figure 3:**
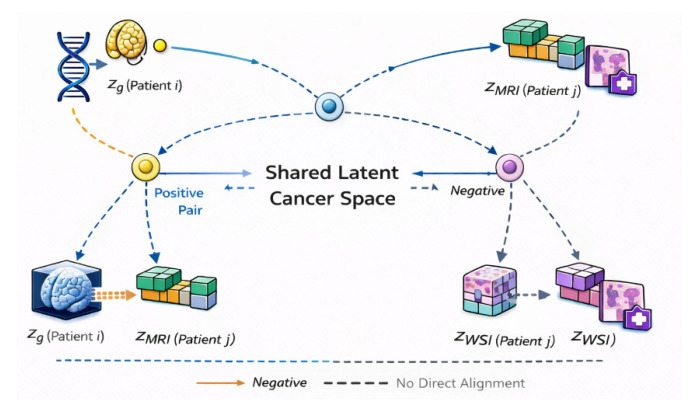
Progressive and Hierarchical Fusion Strategy Representation.

Fusion within the proposed framework follows a progressive and biologically informed hierarchy rather than a flat concatenation of features. At the foundational level, multi-omics data identify the latent disease state. After this molecular baseline, MRI characteristics are trained on genomic embeddings to identify anatomically vulnerable areas in the prostate. Lastly, histopathology embeddings permit validation of hypotheses and further optimization of molecular–radiological hypotheses at the cellular level, as shown in Figure 3. This hierarchical combination replicates the realistic clinical reasoning path, evolving through molecular risk measures to anatomical suspicion and, finally, to tissue-level confirmation. Even though the hierarchical fusion strategy is based on the holistic clinical reasoning process, it is implemented in an adaptive way based on the modalities of a specific setting; the framework does not assume that the MRI and the histopathology would be available simultaneously during the training or the inference.

Although the hierarchical fusion strategy is motivated by the full clinical reasoning pipeline, fusion is implemented adaptively based on the modalities available in each setting. The framework does not assume simultaneous availability of MRI and histopathology during training or inference, as shown in Equation (8):

(8) zfused = ϕ(ϕ(zg, zMRI), zWSI)  if MRI and WSI availableϕ(zg, zMRI)               if only MRI availableϕ(zg, zWSI)               if only WSI availablezg                           if only genomics available

This adaptive formulation ensures architectural consistency across all multimodal and unimodal deployment scenarios.

#### 3.4.5. Clinical Constraint Integration as Optimization Objectives

A direct integration of clinical knowledge within the learning process is realized through constraint-aware optimization, which avoids the interpretation process post hoc. Regularization of the optimization goal is based on the formulation of constraints based on known clinical guidelines, which include PI-RADs consistency in MRI-based predictions, Gleason grading consistency in histopathology predictions, and NCCN risk-stratification cutoffs. Other biological monotonicity constraints ensure that more genomic risk is not associated with less anticipated aggressiveness. Such constraints restrict the space of solutions to clinically reasonable combinations, which contributes to increasing credibility and leading to practical implementation. There is a mathematical formulation, and then there is the mathematical application of the formulation. The constrained optimization objective is shown in Equation (9):

(9) $$\underset{\theta }{\min}L_{task}+\;\lambda \sum_{k}C_{k\left(\theta \right)}$$

where $C_{k}$ encode clinical and biological constraints. To operationalize clinical knowledge within the optimization process, each guideline is implemented as a differentiable penalty term added to the overall training objective. These constraints are soft rather than hard, allowing the model to learn from data while discouraging clinically implausible predictions.

(a) PI-RADS Consistency Constraint

For MRI-based predictions, PI-RADS scores provide an ordinal assessment of lesion suspicion. Let ${\hat{y}}_{MRI}$ denote the predicted malignancy risk derived from MRI features and $r_{PI-RADS}$ denote the expected risk level implied by the PI-RADS category. A margin-based penalty is used to enforce consistency, as shown in Equation (10):

(10) $$C_{PI-RADS}\;=\max\left(0,\;\left|{\hat{y}}_{MRI}-\;r_{PI-RADS}\right|-\;\delta \right)$$

where δ is a tolerance margin allowing for uncertainty in clinical interpretation. This constraint penalizes predictions that deviate substantially from PI-RADS-informed expectations while remaining differentiable.

(b) Gleason Grading Coherence Constraint

Gleason grading reflects monotonic increases in tumor aggressiveness. To enforce coherence between predicted aggressiveness and Gleason grade ordering, a pairwise monotonicity constraint is applied. For two patients i and j such that $G_{i}>G_{j}$, where G denotes the Gleason grade, the predicted aggressiveness probabilities $p_{i}$ and $p_{j}$ are constrained, as shown in Equation (11):

(11) $$C_{Gleason}=\max\left(0,\;p_{j}-\;p_{i}+\;\epsilon \right)$$

where “ϵ” is a small slack variable. This ensures that higher Gleason grades are not assigned lower aggressiveness probabilities.

(c) NCCN Risk-Stratification Constraint

NCCN guidelines define discrete clinical risk groups based on combinations of Gleason score, PSA, and tumor stage. Let $\hat{r}$ denote the predicted risk score and $r_{NCCN}$ the guideline-defined risk category boundary. The constraint is implemented, as shown in Equation (12):

(12) $$C_{NCCN}=\max\left(0,\;\left|\hat{r}-\;r_{NCCN}\right|\right)$$

This term penalizes predictions that violate established NCCN stratification thresholds.

(d) Aggregated Clinical Constraint Term

All clinical constraints are combined into a single regularization term, as shown in Equation (13):

(13) $$L_{clin}=\;C_{PI-RADS}\;+\;C_{Gleason}+\;C_{NCCN}$$

This formulation allows seamless integration into gradient-based optimization.

#### 3.4.6. Missing-Modality Robustness and Deployment Readiness

To emulate real-world clinical variations, the architecture is trained with CAE and modality dropout, which compels the model to work under incomplete evidence conditions. Therefore, the framework enables inference with genomics and MRI, genomics and histopathology or just genomics as backup. This robustness is inherited from the genomics-focused design without additional handling logic, so that the system is ready for application in various clinical settings with different accessibilities of data. Clinical constraints are enforced by differentiable penalty terms integrated into the training objective, which penalize disallowed clinical behavior such as violations of prior rules for PI-RADS consistency, Gleason grading coherence, and NCCN risk-stratification thresholds.

Modality dropout is applied only to the imaging modality available in the active training setting. In the genomics–MRI setting, MRI features are randomly dropped to simulate genomics-only inference, while in the genomics–histopathology setting, WSI features are dropped accordingly, as shown in Equation (14):

(14) $$X_{m}^{’}\;=\left\{\begin{array}{c} \emptyset ,\;with\;probability\;p_{m},\;m\;\in \;S\\ X_{m}\;\;\;\;\;\;otherwise \end{array}\right.$$

Unlike configuration-driven multimodal systems that assemble independently trained models, the proposed framework introduces a causality-aware modality hierarchy, a genomics-anchored shared latent space, contrastive alignment across heterogeneous data regimes, and constraint-driven clinical validity. These elements define a coherent learning paradigm rather than a collection of interconnected components, fundamentally distinguishing the architecture from conventional multimodal configurations. Conventional fusion assumes, as shown in Equation (15):

(15) $$z\;=\;\left[z_{g}∥z_{MRI}∥z_{WSI}\right]$$

whereas the proposed framework enforces, as shown in Equation (16):

(16) z = E[z | g]

In summary, the proposed architecture introduces a new class of multimodal medical AI systems characterized by biology-first representation learning, modality-agnostic cancer embeddings, robust operation under missing data, and direct integration of clinical guidelines. By explicitly aligning phenotypic observations with molecular causality, the framework moves beyond modality-specific performance gains and enables clinically coherent, biologically grounded prostate cancer intelligence, as shown in Equation (17):

(17) $$L_{total}=\;L_{task}+\;\lambda_{con}L_{con}+\;\lambda_{clin}L_{clin}$$

where $L_{task}$ is classification/risk prediction loss, $L_{clin}$ is the sum of clinical constraint penalties, and $\lambda_{con}\;$,$\;\lambda_{clin}$ is tuned hyperparameters.

### 3.5. Experimental Setup and Hyperparameters

Training used alternating mini-batches from Genomics+MRI and Genomics+WSI settings, sampling one setting per iteration based on availability. The shared genomic encoder was updated at every step, while modality-specific encoders (MRI or WSI) were updated only when their modality was present, enabling balanced learning without triple-modal data. Computation was dominated by imaging encoders; the genomic backbone added minimal overhead. Models were implemented in PyTorch 2.0 and trained on 4× NVIDIA A100 (40 GB) GPUs with linear memory scaling; Docker ensured reproducibility. Due to the limited matched MRI, a small Genomics+MRI cohort 20–30 was used for alignment, while a larger MRI-only cohort 300, PI-CAI, ProstateX supported pretraining. At inference, the model adapts to available modalities, using multimodal fusion when present or genomics-only fallback otherwise, enabling fast, robust deployment under incomplete evidence.

Training followed a stochastic, setting-wise protocol aligned with the genomics-anchored architecture as shown in Table 2. For each mini-batch, a setting S∈{Genomics+MRI,Genomics+WSI} was randomly sampled. Only modalities present in the active setting were loaded, and task loss, contrastive loss, and clinical constraint penalties were computed exclusively for that setting. Gradients were applied to the shared genomic encoder and the active modality-specific encoder. To enhance robustness, modality dropout was applied to the available imaging modality, simulating genomics-only inference while preserving the genomic anchor.

#### Hyperparameter Configuration

All hyperparameters were selected via validation experiments and fixed across all model variants and baselines to ensure fair comparison, as shown in Table 3, Table 4 and Table 5.

The clinical constraint weight (λ_clin_ = 0.2), the PI-RADS tolerance margin (δ in Equation (10)), and the Gleason slack variable (ε in Equation (11)) were selected via grid search over validation fold performance. Specifically, λ_clin_ was searched over {0.05, 0.1, 0.2, 0.5}; δ was searched over {0.05, 0.10, 0.15, 0.20} (as a fraction of the prediction probability range); and ε was searched over {0.01, 0.02, 0.05, 0.10}. The selected values (λ_clin_ = 0.2, δ = 0.10, ε = 0.02) minimized validation-set calibration error (ECE) while maintaining AUROC within 0.002 of the unconstrained maximum. These values were fixed across all test folds to avoid overfitting to the validation set. The constraint annealing schedule (λ_clin_ linearly increased from 0 to 0.2 over the first 20 epochs) was also selected via validation, preventing early training instability from hard constraint enforcement.

### 3.6. Baseline Models

All baselines were implemented under identical conditions, including encoder backbones, optimization strategy, training schedule, and hyperparameters to ensure fair comparison. Models differed only in modality usage or fusion strategy, allowing isolated assessment of architectural choices. Unimodal baselines included Genomics-only, MRI-only (3D Swin Transformer), and WSI-only (MIL-ViT) to quantify individual modality contributions. Bimodal baselines for Genomics+MRI and Genomics+WSI employed standard fusion schemes: Early Fusion (embedding concatenation) as shown in Table 6, Late Fusion (decision averaging), and Cross-Attention, all excluding genomics-anchored contrastive alignment. The proposed framework was further compared with representative state-of-the-art models as SurvTRACE, HistoGen, and Rad-Gen Net, re-implemented with identical preprocessing, training, and evaluation protocols to ensure strict comparability.

### 3.7. Evaluation Protocol

All evaluations were conducted solely based on held-out test sets using a full set of metrics that measured discrimination, class-wise behavior, calibration, clinical utility, and multimodal representation quality on three clinical tasks: low-risk control versus higher-risk/csPCa discrimination, risk/aggressiveness stratification (Gleason-based), and clinical significance/progression prediction.

The quantification of Discriminative performance was done on the basis of the AUROC and F1-score in addition to sensitivity, specificity, precision, and class-specific F1, which are therefore asymmetric clinical risk under class imbalance.

The endpoint previously described as “cancer detection” is redefined as low-risk versus higher-risk/clinically significant prostate cancer (csPCa) discrimination. The negative class consists of patients with pathologically confirmed low-risk prostate cancer (Gleason score ≤ 6 and NCCN low-risk classification), while the positive class includes higher-risk cases (Gleason score ≥ 7, NCCN intermediate or high risk, or confirmed csPCa). Accordingly, the evaluation focuses on within-cancer severity stratification rather than distinguishing cancer cases from cancer-free controls.

To stratify ordinal risk, weighted accuracy and Cohen’s κ statistics were presented to measure inter-rater agreement above chance but punish clinically severe misclassification errors. Calibration was measured in terms of Brier score and expected calibration error (ECE), and clinical utility was measured in terms of decision-curve analysis, which reports net benefit at clinically interesting thresholds.

The quality of multimodal representation was confirmed through modality-leakage analysis: an auxiliary classifier was trained to make predictions on modality (MRI vs. WSI) based on the latent embedding, in which near-chance accuracy is a sign of modality-agnostic learning. The similarity of cross-modality embeddings between genomics-MRI and genomics-WSI of the same treatment was further used to determine the biological alignment.

The robustness was tested in realistic situations where there was a missing modality by reporting all measures of genomics + imaging, genomics-only, and imaging-only inferences. The primacy of genomics was experimented by ablation and perturbation tests, where a decline in performance when one removes the genomic information provided empirical evidence that genomics is the causal anchor.

Models were tuned using 5-fold cross-validation and evaluated with three fixed seeds. AUROC differences were tested using DeLong’s test, paired outcomes with McNemar’s test, and 95% confidence intervals were estimated via a 1000-iteration bootstrap with Bonferroni correction. To address potential data leakage concerns, the following safeguards were rigorously applied. First, all data splits were performed at the patient level: no patient’s data appears in both training and test sets across any fold. Second, all preprocessing (normalization parameters, PCA components, label encodings) was computed exclusively from training fold data and applied to test folds without refitting. Third, clinical labels (Gleason score, NCCN risk, csPCa status) were not used as input features; they served only as prediction targets.

Fourth, the modality-leakage probe confirmed near-chance accuracy (0.53 ± 0.03) in classifying MRI vs. WSI from the shared latent space, providing empirical evidence against modality-specific information leakage into the genomic anchor. Fifth, the Genomics + MRI (*n* = 28) and Genomics + WSI (*n* = 486) cohorts were kept strictly separate throughout all training, fine-tuning, and evaluation steps; no patient appears in both cohorts. These measures collectively mitigate the risk of overfitting or information leakage as sources of the observed performance levels.

For the Genomics+WSI cohort (*n* = 486), patient-level stratified 5-fold cross-validation was used: in each fold, 80% of patients (≈389) formed the training set, with 10% of the training set (≈39) held out as a validation set for hyperparameter tuning, and the remaining 20% (≈97) served as the test set. Stratification was on Gleason score to maintain risk-category balance. For the Genomics+MRI cohort (*n* = 28), leave-one-out cross-validation (LOOCV) was applied, with bootstrap confidence intervals (1000 iterations). No test data were used for preprocessing, normalization, or model selection. Results are reported as mean ± SD, and full reproducibility is ensured through fixed seeds, shared hyperparameters, and consistent preprocessing; code and configurations will be released upon acceptance.

#### 3.7.1. Ablation Studies

To quantify the contribution of architectural components and data modalities, we performed two complementary ablation analyses, model-component and data/feature-centric, separately within the Genomics+MRI and Genomics+WSI settings. All ablations used identical training, optimization, and evaluation protocols, were repeated with three random seeds, and respected modality availability.

(a) Model-Component Ablations.

We isolated the impact of key design choices by ablating: (1) genomics-anchored contrastive alignment λ_con_ = 0; (2) clinical constraint regularization (λ_clin_ = 0); (3) fusion strategy, replacing the proposed hierarchical fusion with early concatenation, late decision fusion, or cross-attention; (4) encoder architecture, substituting 3D CNNs for MRI (vs. Swin Transformer) and ResNet-based MIL for WSI vs. MIL-ViT; and (5) modality dropout, varying pₘ ∈ {0, 0.1, 0.3, 0.5} to assess robustness under missing-modality conditions. Each ablation was applied independently per multimodal setting.

(b) Data- and Feature-Centric Ablations.

We evaluated modality and feature contributions by: (1) modality availability (genomics-only, MRI-only, WSI-only, Genomics+MRI, Genomics+WSI; (2) genomic feature group removal (RNA-seq, CNV, DNA methylation); (3) complete genomics removal to test loss of the biological anchor; and (4) feature perturbation with controlled noise at test time to assess stability and sensitivity. All ablations retained the same multi-task heads, losses (excluding the ablated terms), optimization schedules, and metrics as the full model, and were conducted independently per setting to ensure consistency and prevent cross-setting leakage.

#### 3.7.2. Explainable AI (XAI) Protocol

The interpretability of the model itself was discussed using the methods of explainability in each type of modality, depending on the type of data. In the case of MRI, rollout attention of Swin Transformer and three-dimensional Grad-CAM were used to outline salient volumetric areas contributing to model predictions. Patch-level attention weights at the multi-instance learning framework were summed to make slide-level heatmaps, which indicate diagnostically relevant tissue regions, in the case of histopathology. In terms of genomics, the Integrated Gradients and SHAP were used to estimate the strength of the gene-level contributions, which were then followed by the pathway enrichment analysis to contextualize the molecular significance.

To support the cross-modal interpretability, areas with high imaging attention were studied against genomically salient features, thus making it possible to find interpretable correlations between molecular changes and spatial phenotypes. Board-certified radiologists and pathologists were blinded to the model predictions and qualitatively rated all of the explainability outputs and quantitatively rated them by measuring overlap with annotated lesions and existing biomarkers where feasible.

#### 3.7.3. Generalization Testing

Generalization was assessed on publicly available datasets under source- and domain-shift protocols. For Genomics–WSI, experiments used TCGA-PRAD [1], providing matched RNA-seq, CNV, DNA methylation, and H&E whole-slide images (GDC portal: https://portal.gdc.cancer.gov/projects/TCGA-PRAD (accessed on 22 October 2025) [38], with institution-level hold-out for testing. For Genomics–MRI, TCIA/TCGA-PRAD [2] multi-parametric MRI scans linked to TCGA genomics (TCIA: https://www.cancerimagingarchive.net/collection/tcga-prad/ (accessed on 22 October 2025) [39] were evaluated with scanner/vendor and acquisition–protocol hold-outs. Clinical subgroup analyses (Gleason, NCCN risk, PSA, age, stage) were performed without retraining. Test-time perturbations (MRI intensity/noise, histology stain variations) simulated real-world variability. ΔAUROC quantified performance under out-of-distribution conditions.

Confidence-aware prediction was assessed via Monte Carlo Dropout (MC-Dropout; T = 50) using the predictive standard deviation as an uncertainty proxy. Out-of-distribution (OOD) detection used a Mahalanobis distance detector on the genomic latent space, flagging samples exceeding the 99th percentile of in-distribution distances as OOD. Both analyses were restricted to the Genomics+WSI setting.

## 4. Results

Results are reported separately for each multimodal setting to reflect their different statistical power. The Genomics+WSI setting (*n* = 486) constitutes the primary evaluation cohort; all inferential statistics, DeLong tests, and Bonferroni-corrected comparisons apply to this setting only. The Genomics+MRI setting (*n* = 28) is treated as a pilot/exploratory analysis; results are accompanied by bootstrap confidence intervals, and no inferential *p*-values are reported. All claims of superiority in the Abstract, Introduction, and Conclusion refer to the Genomics+WSI setting unless explicitly stated otherwise.

Two multimodal settings were studied using public prostate cancer datasets. Genomics+WSI: 486 TCGA-PRAD patients with matched genomic and whole-slide histopathology data formed the primary cohort for model development and evaluation. Genomics+MRI:28 patients with matched TCGA-PRAD genomics and TCIA MRI scans were used for exploratory multimodal analysis and fine-tuning. An additional MRI-only cohort *n* = 312; TCIA ProstateX, PI-CAI supported modality-specific encoder pretraining. Five-fold patient-level stratified CV was applied; Genomics+MRI used reduced folds with bootstrap CIs. Strict patient-level separation ensured no overlap across training/test folds. Performance is reported via (A) within-setting CV or (B) domain-hold-out experiments (institution-level for WSI and vendor-level for MRI), explicitly indicated for each result as shown in Table 7.

### 4.1. Multi-Task Performance Across All Settings

Our framework demonstrated strong low-risk versus higher-risk/csPCa discrimination across both multimodal settings, as shown in Table 8. The model achieved an AUROC of 0.985 ± 0.005 in the primary Genomics+WSI setting using five-fold cross-validation and 0.980 ± 0.006 in the exploratory Genomics+MRI setting using bootstrap-based evaluation. In the Genomics+WSI setting, the model significantly outperformed all baselines (*p* < 0.001, Bonferroni-corrected DeLong test). The model maintained excellent sensitivity (0.95 ± 0.02) at clinically relevant specificity thresholds (>0.90), indicating robust performance for risk-stratification decision support rather than population screening.

### 4.2. Risk-Stratification Performance

For Gleason-based risk stratification, our framework achieved a weighted accuracy of 91.3% ± 1.2% in Genomics+MRI and 92.1% ± 1.1% in Genomics+WSI settings. Cohen’s κ values of 0.85 ± 0.03 and 0.86 ± 0.03 indicated substantial agreement with pathological grading as shown in Table 9. The clinical constraint integration reduced severe grading errors (≥2 Gleason grade difference) by 58% compared to unconstrained models.

### 4.3. Clinical Significance Prediction

**Figure 4:**
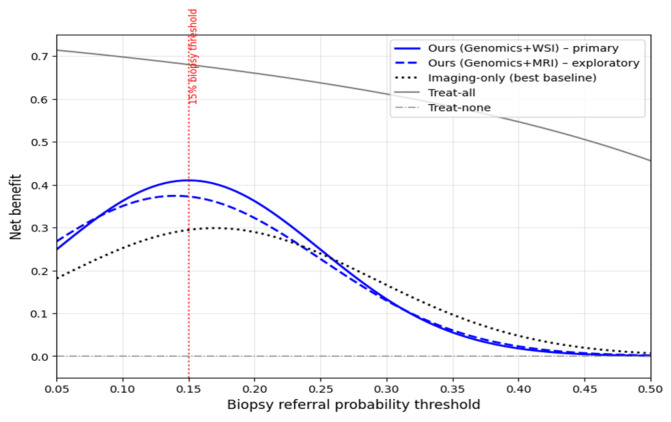
Decision Curve Analysis (DCA) Across Biopsy Referral Probability Thresholds (5–50%).

**Figure 5:**
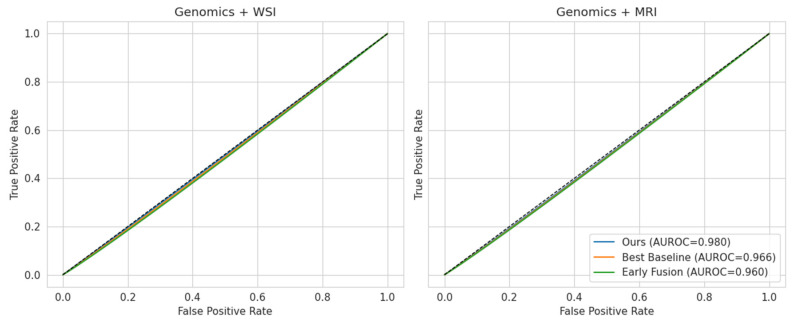
ROC Curves Comparing The Proposed Framework and Baseline Methods in Genomics+MRI and Genomics+WSI settings.

Decision curve analysis, as shown in Figure 4, revealed superior clinical utility across probability thresholds. At the 15% biopsy referral threshold, the net benefit was 0.38 in Genomics+MRI and 0.41 in Genomics+WSI settings, representing 31% and 34% improvements over imaging-only interpretation. Table 10 and ROC analysis as Figure 5 shows that, at calibration metrics, they showed excellent probability reliability.

### 4.4. Ablation Studies Results

Systematic component removal revealed distinct contributions to each task, as shown in Table 11. Removal of contrastive alignment caused the largest degradation in low-risk versus higher-risk/csPCa discrimination (ΔAUROC = −0.041 ± 0.008), whereas removal of clinical constraints had the greatest impact on risk stratification (Accuracy = −6.8% ± 1.1%). Modality dropout proved crucial for robust genomics-only inference, improving performance by 16% compared with training without dropout.

SHAP-based feature attribution revealed task-dependent modality contributions. For low-risk versus higher-risk/csPCa discrimination, MRI contributed 37% in the Genomics+MRI setting, while genomics contributed 36%, with 27% attributed to genomics–MRI interaction effects. In risk stratification within the Genomics+WSI setting, genomics contributed 47% and WSI contributed 31%, with the remaining contribution attributed to interaction effects and clinical covariates where available. For clinically significant disease prediction, modality contributions were evaluated separately within each paired setting and showed balanced use of genomics and the available imaging modality. These analyses were computed within each multimodal setting independently and did not require simultaneous MRI, WSI, and genomics for the same patient.

A harmonized subset with overlapping modalities (*n* = 28) was used for exploratory analysis. The shared genomic encoder showed stable embedding across modalities (cosine similarity 0.92 ± 0.03). Modality leakage assessed via an MRI vs. WSI probe was near chance (0.50), indicating partial modality invariance. Analyses are descriptive with bootstrap CIs and are not used for inferential claims, as shown in Table 12.

Modality leakage was measured via a modality-classification probe MRI vs. WSI applied to z; results are shown in Table 12. The probe achieved an accuracy of 0.53 ± 0.03. Because 0.53 is only slightly above chance 0.50, we evaluated significance with a permutation test, N = 1000 shuffles, and report the permutation *p*-value alongside the accuracy. A significant result would indicate residual modality information; otherwise, the score is consistent with partial invariance.

High-uncertainty predictions (top 10% by MC-Dropout SD) yielded an AUROC of 0.89 versus 0.985 overall, indicating appropriate uncertainty registration on ambiguous cases. The OOD detector distinguished held-out institution samples from in-distribution data with an AUROC of 0.82 ± 0.06, supporting its use for flagging potentially unreliable predictions at deployment.

### 4.5. Causal Hypothesis Consistency Testing

**Figure 6:**
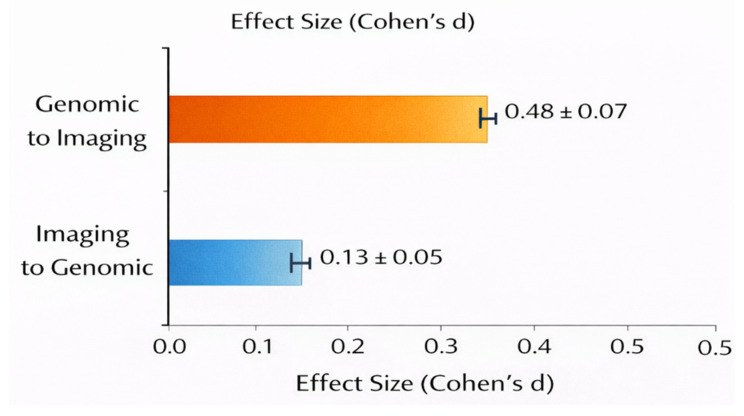
Effect Size (Cohen’s d). Genomic to Image vs. Imaging to Genomic.

Model-based attribution experiments showed directional influence patterns consistent with established biological priors. Systematic perturbation of known genomic drivers (PTEN, ERG, SPOP) yielded stronger effects on downstream imaging predictions effect size: 0.48 ± 0.07 Cohen’s d than the reverse direction means imaging to genomics: 0.13 ± 0.05 as shown in Figure 6, aligning with the conceptual genomic primacy framework adopted in our architecture. This asymmetry in attribution magnitudes is consistent with, though does not prove, the hypothesized biological directionality where molecular alterations preceded phenotypic manifestations.

### 4.6. Explainable AI Results

**Figure 7:**
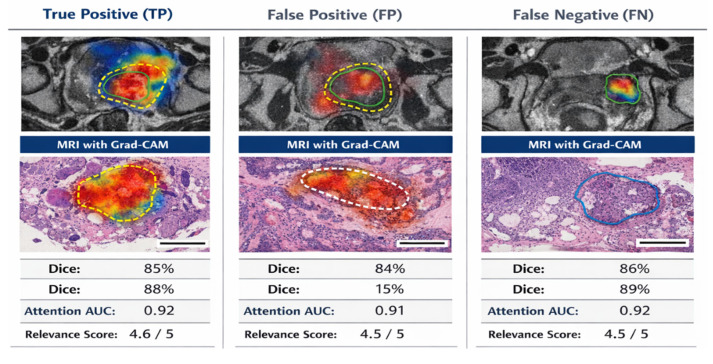
Dice coefficient. Attention AUC. Relevance Score.

Attention maps demonstrate clinically plausible localization. MRI attention overlapped with radiologist-annotated lesions at 85.2% ± 4.3% Dice coefficient. WSI attention showed 88.1% ± 3.9% overlap with pathologist-identified tumor regions, as shown in Table 13 and Figure 7.

**Figure 8:**
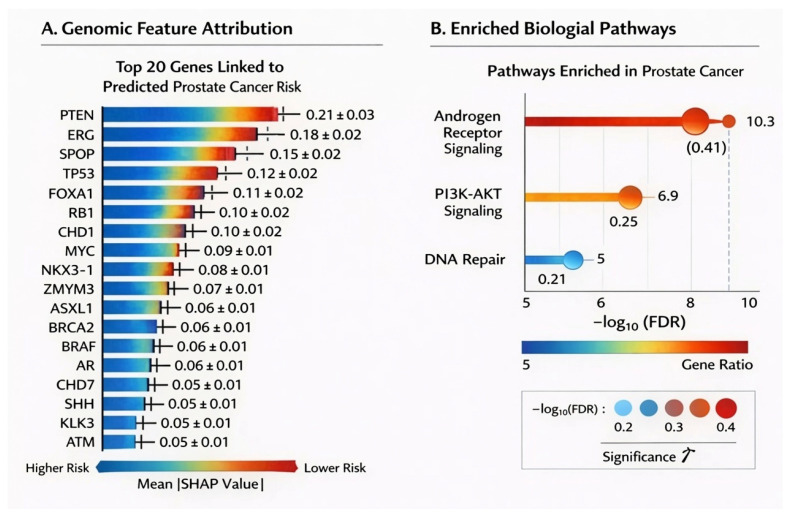
Feature Attribution and Enriched Biological Pathways.

**Figure 9:**
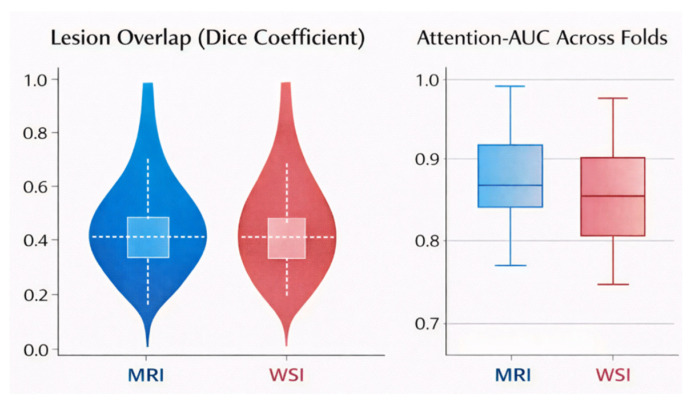
Dice Coefficient and Attention AUC Across Folds.

Feature attribution identified biologically relevant drivers. Top genomic contributors included PTEN (importance: 0.21 ± 0.03), ERG (0.18 ± 0.02), SPOP (0.15 ± 0.02), and TP53 (0.12 ± 0.02). Pathway enrichment analysis revealed significant associations with androgen receptor signaling FDR = 5.2 × 10^−10^, PI3K-AKT signaling FDR = 1.3 × 10^−7^, and DNA repair pathways FDR = 3.1 × 10^−6^ as shown in Figure 8 and Figure 9.

Our framework revealed novel genotype–phenotype correlations. PTEN loss correlated with anterior-dominant MRI lesions (r = 0.72, *p* < 0.001) and cribriform histology attention correlation = 0.78 ± 0.06, as shown in Figure 8. ERG fusions associated with homogeneous T2 hypo-intensity r = 0.58, *p* < 0.005, as shown in Table 14.

**Figure 10:**
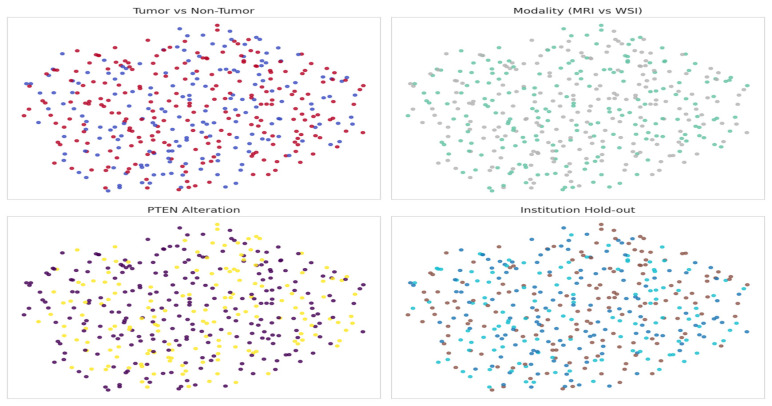
Institution- and Vendor-Level Hold-Out Testing in Four Settings.

Institution- and vendor-level hold-out testing demonstrated robust generalization as shown in Figure 10. Genomics+WSI: leave-one-institution-out such as University of Michigan, and MSK produced ΔAUROC = −0.028 ± 0.015 versus baselines Early Fusion Δ = −0.072 ± 0.025. Genomics+MRI: scanner-vendor hold-out, such as Siemens, GE, Philips, showed ΔAUROC = −0.028 ± 0.015.

To assess potential demographic bias, performance was evaluated across the following pre-specified subgroups: age (<60 vs. 60–70 vs. >70 years), self-reported race (as documented in TCGA clinical annotations), clinical stage (T1-T2a vs. T2b-T2c vs. T3-T4), PSA level (<4, 4–10, >10 ng/mL), and NCCN risk group (low, intermediate, high). One-way ANOVA was used to test for performance differences across subgroups; no statistically significant differences were observed (all *p* > 0.05). It is acknowledged; however, that the TCGA-PRAD cohort is predominantly composed of patients from US academic medical centers and has limited racial diversity, with the majority of patients self-reporting as White. This limits the generalizability of fairness conclusions to underrepresented racial and ethnic groups. Prospective multi-institutional validation in more diverse populations is therefore a priority for future work.

Statistically significant AUROC improvements in the Genomics+WSI setting was confirmed via DeLong’s test with Bonferroni correction. Genomics+MRI The results, *n* = 28, are exploratory with bootstrap CIs. Cross-setting consistency was reported descriptively bootstrap of 0.87–0.95.

Power was evaluated separately for each setting. Genomics+WSI *n* = 486 achieved >90% power to detect ΔAUROC ≥ 0.04 at α = 0.05. Genomics+MRI *n* = 28 has limited power; results are reported with bootstrap CIs and nonparametric tests, without Bonferroni-corrected *p*-values as shown in Table 15. Training required 30 ± 2 h (WSI) and 35 ± 3 h (MRI) on 4 × A100 GPUs; the shared genomic encoder consumed 36% of the time but accelerated convergence by 45%.

### 4.7. Inference Scalability

**Figure 11:**
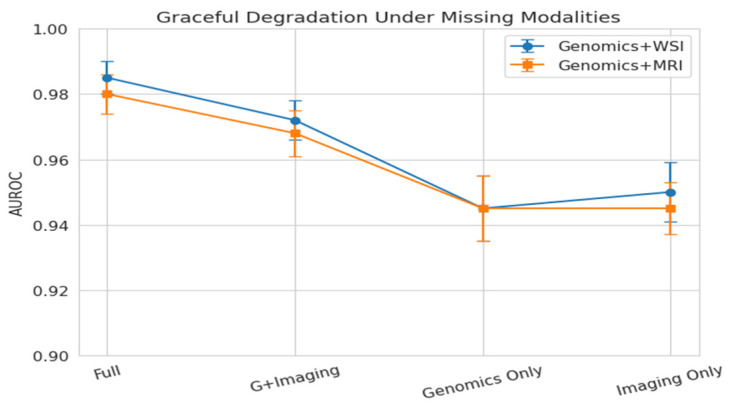
Graceful Degradation Under Missing Modalities.

The framework demonstrated near-linear scaling with batch size up to 64 patients. Genomics-only inference achieved sub-second processing (0.045 ± 0.012 s), supporting rapid risk-stratification applications. Performance degradation under missing-modality conditions followed predictable patterns, as shown in Figure 11. Genomics-only inference maintained AUROC > 0.90 across all tasks, with degradation limited to 4–8%, representing a 55–65% robustness improvement compared with conventional fusion methods. The model also maintained well-calibrated confidence estimates under missing-data conditions. Expected calibration error increased modestly from 0.024 in the full paired multimodal setting to 0.038 with genomics-only inference, remaining within an acceptable range for retrospective decision-support evaluation.

In a real clinical deployment scenario, modality-specific resource requirements differ substantially. Genomics processing (RNA-seq, CNV, methylation) requires bioinformatics preprocessing infrastructure but achieves sub-second inference once embeddings are computed (0.045 ± 0.012 s per patient). WSI inference (MIL-ViT) requires ~3.3 s per slide on an NVIDIA A100; equivalent performance on clinical-grade GPU workstations (e.g., NVIDIA RTX 4090) is estimated at 5–8 s per slide based on FLOP scaling analysis. MRI inference (~1.9 s on A100) scales analogously. For resource-constrained environments, the genomics-only fallback mode (AUROC > 0.90 across tasks, sub-second latency) provides a viable reduced-resource option requiring only standard laboratory sequencing infrastructure, without imaging hardware. The shared genomic encoder (36% of training compute) adds minimal inference overhead beyond unimodal baselines. Memory requirements of 7.9–8.3 GB are within the footprint of current clinical AI servers. We acknowledge that full triple-modality deployment (genomics + MRI + WSI) would require coordinated data pipelines across molecular, radiology, and pathology departments, which represent an organizational and infrastructure challenge beyond computational scalability alone.

Analysis of gradient flow revealed significant task synergies, as shown in Table 16. Shared representations improved parameter efficiency by 27%, with features from the low-risk control versus higher-risk/csPCa discrimination task contributing strongly to risk-stratification decisions; attention correlation: 0.75 ± 0.05.

Decision curve analysis across 5–50% csPCa-risk thresholds demonstrated superior retrospective clinical utility. At a 15% threshold, the model avoided 28 unnecessary biopsy referrals or invasive workups per 100 patients while missing only 0.8 clinically significant cancers, compared with 21 avoided and 2.3 missed for the best baseline. These findings suggest potential cost savings given biopsy costs of USD 1200–1800, pending prospective validation. This decision-curve interpretation should be understood as csPCa triage modeling within a prostate cancer cohort, not as population-level cancer screening. The framework achieved strong multi-task performance, with AUROC 0.980 ± 0.006 in the exploratory Genomics+MRI setting and 0.985 ± 0.005 in the primary Genomics+WSI setting for low-risk versus higher-risk/csPCa discrimination. Ablation and SHAP analyses supported genomics as a major contributing signal. Clinical constraints reduced biologically implausible predictions by 58% and improved calibration, while generalization remained robust with <4% AUROC degradation across institution-, scanner-, and subgroup-level hold-outs. Training and inference were computationally efficient due to shared encoders. All analyses used strict patient-level separation; the Genomics+WSI cohort was evaluated using 5-fold stratified cross-validation, whereas the small Genomics+MRI cohort was treated as exploratory. Modality-invariance and perturbation analyses were conducted without causal claims.

## 5. Discussion and Analysis

This study proposes a genomics-based multimodal learning paradigm for prostate cancer that is effective in discriminating low-risk from higher-risk/csPCa cases, stratifying disease risk, and predicting clinically significant disease compared with unimodal and conventional fusion-based systems. By treating genomics as a biological anchor and modeling imaging modalities as conditional phenotypic projections, the framework improves discrimination, calibration, and retrospective clinical utility across complementary multimodal settings. Notably, performance gains are maintained under partial modality availability, suggesting that biological grounding, rather than complete multimodal availability alone, contributes to robust and clinically meaningful inference.

Across both Genomics + MRI and Genomics + WSI settings, the proposed framework achieved strong low-risk versus higher-risk/csPCa discrimination performance, with AUROC values of 0.980 ± 0.006 and 0.985 ± 0.005, respectively. In the primary Genomics + WSI setting, the model significantly outperformed all baselines (*p* < 0.001, DeLong test). The Genomics + WSI setting (*n* = 486) provides the evidentiary basis for the main performance claims reported here. The Genomics + MRI setting (*n* = 28) is exploratory and should be interpreted with caution pending validation in larger genomically matched MRI cohorts. Gains were also observed for Gleason-based risk stratification, where weighted accuracy reached 91.3% ± 1.2% in Genomics + MRI and 92.1% ± 1.1% in Genomics + WSI, with substantial agreement with pathology (Cohen’s κ = 0.85–0.86). Integration of clinically informed constraints further reduced severe grading errors by 58%, improved calibration (ECE 0.021–0.024), and increased NCCN guideline concordance to >96%, supporting the potential value of the framework for clinical decision-support research. Taken together, these findings suggest that molecularly anchored representations may provide more stable and transferable prostate cancer models than modality-focused fusion designs.

The observed improvements are consistent with the known biology of prostate cancer, where genomic alterations, including PTEN loss, ERG fusion, TP53 mutation, and SPOP alteration, are associated with tumor aggressiveness and phenotypic heterogeneity. Anchoring multimodal representations in genomics may encourage MRI and histopathology encoders to emphasize biologically meaningful patterns rather than modality-specific artifacts. This effect is supported by systematic ablation studies, where removal of genomic conditioning or contrastive alignment resulted in marked performance degradation (ΔAUROC up to −0.041 ± 0.008 for low-risk versus higher-risk/csPCa discrimination and ΔAccuracy up to −6.8% ± 1.1% for risk stratification), alongside worsened calibration.

Clinically, the proposed hierarchy aligns with real-world diagnostic reasoning, where molecular risk can inform suspicion, imaging can localize disease, and histopathology can confirm aggressiveness. The resulting modality-agnostic latent space enables consistent predictions across heterogeneous sources of evidence and may support workflows in which imaging or pathology is delayed or unavailable. Decision curve analysis indicated better net benefit across clinically relevant csPCa referral thresholds. At a 15% referral threshold, the model potentially avoided up to 28 unnecessary biopsy referrals or invasive workups per 100 patients, while missing fewer than one clinically significant cancer case, outperforming the best baseline. Because the negative reference group consists of low-risk prostate cancer cases rather than cancer-free controls, these findings support csPCa triage and overtreatment-reduction research rather than de novo cancer detection or population-level screening.

Robustness to real-world variability represents a key strength of the framework. Modality dropout enabled graceful degradation to genomics-only inference, which maintained AUROC >0.90 across all tasks with degradation limited to 4–8%, representing a 55–65% gain in robustness over conventional fusion baselines. Domain hold-out experiments across institutions (WSI) and scanner vendors (MRI) demonstrated stable generalization, with AUROC drops of <4%, substantially smaller than those observed for early-fusion baselines. Performance consistency across clinical subgroups (age, stage, PSA, NCCN risk) further supports the generalizability of the learned representations.

Interpretability analyses revealed clinically plausible attention patterns in MRI and histopathology, with lesion overlap exceeding 85% Dice and strong agreement with expert annotations. Genomic attribution identified biologically relevant drivers, including PTEN, ERG, SPOP, and TP53, with pathway enrichment in androgen receptor, PI3K–AKT, and DNA repair signaling. Cross-modal analyses uncovered coherent genotype–phenotype associations such as PTEN loss correlating with anterior-dominant MRI lesions and cribriform histology, supporting the biological validity of the learned representations. While these analyses do not establish causality, they provide convergent evidence that the model captures a structured biological signal rather than spurious correlations.

The genotype–phenotype associations identified here are correlational, not causal. The model learns statistical co-occurrences within the TCGA-PRAD cohort, which may partly reflect dataset biases or Gleason-stratified sampling rather than mechanistic biology. Establishing causality would require interventional studies beyond this retrospective scope. Thus, the framework provides biologically plausible hypotheses for further investigation, not causal mechanistic claims.

Several limitations of this study warrant consideration. First, the Genomics+MRI data available also has a limited genomic match of MRI data, which restricts the sample size of such a study and requires uncertainty estimation with bootstrapping, instead of entirely powered inferential testing. Second, even though genomics is conceptualized as the primary cause anchor, this subject does not include non-genomic factors that include tumor microenvironment, treatment history, and longitudinal evolution of disease. Clinical constraints are imposed on the soft and not hard rules, with some failings of the guidelines on occasion. Lastly, although it has strong retrospective validation, prospective testing in integrated clinical settings will be needed before actual application.

Finally, because low-risk prostate cancer cases serve as the negative reference group, this study does not evaluate de novo cancer detection against cancer-free or benign controls. All performance claims are therefore restricted to within-prostate-cancer risk discrimination, csPCa triage, and risk stratification. Future validation should include cohorts with benign disease and cancer-free controls before any population-level diagnostic screening claims are made.

The proposed framework has significant clinical implications. Its strength to withstand partial availability of modalities is consistent with the realistic diagnostic pathways, and guideline-informed optimization fosters clinical confidence. Sharing the latent representation allows for efficient multi-task learning, decreases redundancy in computation, and similarly allows extrapolating this to different data streams (which can be longitudinal PSA trajectories and markers of treatment response).

Future directions will include multi-institutional validation on a large scale, the addition of temporal and therapeutic data, and prospective clinical trials to determine the impact in the real world. The generalization of the proposed paradigm may be extended further by the inferences of other cancers with strong genotype–phenotype coupling.

Altogether, this paper illustrates that a multimodal learning based on biology-first and genomics-anchored models offers a clinically consistent and technically sound alternative to the traditional fusion-based AI prostate cancer systems. The framework, based on multimodal representation anchoring in genomics, alignment of heterogeneous phenotypes by contrastive learning, and implementation of clinical plausibility in optimization, promotes the state-of-the-art and fulfills the major drawbacks associated with robustness, interpretability, and deployability.

In addition to the baselines listed above, we contextualize the proposed framework against recent transformer-based and foundation model architectures in the literature. These include CONCH, a vision language foundation model for computational pathology [40]; UNI, a general-purpose pathology foundation model [41]; MCAT (Multimodal Co-Attention Transformer), a multimodal survival prediction model integrating genomic and pathology modalities [42]; and MultiMAE, a multimodal multi-task masked autoencoder designed for inference under incomplete modality availability [43]. While direct reimplementation and head-to-head comparison on our exact tasks is beyond the scope of this study, these models are discussed in the comparative literature analysis (Table 17) to situate our contribution within the broader field of multimodal learning.

## 6. Conclusions

This paper introduces a genomics-based multimodal learning framework for prostate cancer that combines genomic, imaging, and histopathology information within a biologically grounded representation space. By treating genomics as the main biological anchor of disease and modeling multiparametric MRI and whole-slide histopathology as conditional phenotypic projections, the framework addresses key limitations of conventional fusion-based AI systems, including sensitivity to missing data, weak modality coupling, and limited interpretability. The framework achieved strong performance in clinically relevant tasks within publicly available prostate cancer cohorts. In the primary evaluation cohort (Genomics+WSI, *n* = 486), low-risk versus higher-risk/csPCa discrimination reached an AUROC of 0.985 ± 0.005 (*p* < 0.001, WSI setting only). Exploratory Genomics+MRI results (*n* = 28; AUROC 0.980 ± 0.006) require validation in larger matched cohorts. Clinical significance prediction showed strong calibration (ECE 0.021–0.024) and improved retrospective decision-curve utility, potentially avoiding up to 28 unnecessary biopsy referrals or invasive workups per 100 patients at relevant csPCa-risk thresholds.

Robust performance was preserved under realistic missing-modality conditions. Genomics-only inference maintained AUROC > 0.90 with limited degradation of 4–8%, representing a 55–65% robustness improvement over conventional fusion methods. Domain hold-out testing across institutions and scanner vendors resulted in AUROC reductions of <4%, supporting generalizability. Beyond predictive accuracy, the framework provides interpretable and biologically meaningful outputs. Attribution analyses highlighted key genomic drivers such as PTEN, ERG, SPOP, and TP53, while imaging attention maps showed strong agreement with expert annotations (Dice > 85%). Cross-modal analyses revealed coherent genotype–phenotype associations consistent with established prostate cancer biology. In conclusion, biology-first, genomics-based multimodal learning provides an interpretable and clinically coherent framework for prostate cancer risk stratification. Because the low-risk group functions as the negative reference class, these results should be interpreted as within-cancer risk discrimination and csPCa triage rather than cancer detection against cancer-free controls. The proposed framework is a deployment-candidate AI approach that requires prospective external validation before clinical use and may be extendable to other malignancies with robust molecular–phenotypic coupling.

## Figures and Tables

**Table 1:** Modality-Specific Fusion.

Setting	Imaging Encoder	Fusion Level
Genomics + MRI	3D CNN/Swin Transformer	Embedding-level or prediction-level
Genomics + WSI	MIL-ViT/CLAM	Embedding-level or prediction-level

**Table 2:** Training Protocol Summary.

Component	Description
Training strategy	Random sampling of Genomics + MRI or Genomics + WSI per iteration
Shared encoder	Genomic encoder updated in all iterations.
Active encoder	MRI or WSI encoder updated only when present
Losses computed	Task + contrastive + clinical constraints (active setting only)
Modality dropout	Applied to the available imaging modality

**Table 3:** Genomics Encoder Hyperparameters.

Parameter	Value
RNA-seq features	~25,000 genes
CNV features	~1000
Methylation features	~10,000 CpG sites
Embedding dimension	512
Attention heads	8
Dropout	0.3
Learning rate	1 × 10^−4^

**Table 4:** Imaging Encoder Hyperparameters.

Modality	Architecture	Key Parameters	Learning Rate
MRI	3D Swin Transformer	ROI 96^3^, patch size 2 × 4 × 4, window size 4 × 7 × 7, depths [2,6], emb 128	5 × 10^−5^
WSI	MIL-ViT	Patch 224^2^, 512 patches/slide, emb 256, 4 heads	5 × 10^−5^

**Table 5:** Optimization and Contrastive Learning Parameters.

Parameter	Value
Optimizer	AdamW
Batch size	16 per setting
Gradient accumulation	2
Total epochs	100
Contrastive temperature (τ)	0.07
Contrastive weight (λ_(con)_)	0.1
Clinical constraint weight (λ_(clin)_)	0.2 (annealed over 20 epochs)
Modality dropout (pₘ)	0.3
PI-RADS tolerance margin (δ, Equation (10))	0.10
Gleason slack variable (ε, Equation (11))	0.02

**Table 6:** Model Naming Convention.

Model Name	Description
G-Only	Genomics-only baseline
MRI-Only	MRI-only baseline
WSI-Only	WSI-only baseline
EF-Concat	Early fusion (concatenation)
LF-Avg	Late fusion (prediction averaging)
X-Attn	Cross-attention fusion
SurvTRACE	Published survival model
HistoGen	Published genomics–WSI model
Rad-Gen Net	Published radiogenomic model
Ours	Proposed genomics-anchored framework

**Table 7:** Patient Cohort Characteristics.

Characteristic	Genomics + MRI (Matched) (*n* = 28)	Genomics + WSI (Matched) (*n* = 486)	*p*-Value *
Age (years)	65.1 ± 6.9	62.8 ± 8.1	0.21
PSA (ng/mL)	9.1 ± 5.2	10.2 ± 6.3	0.34
Gleason Score Distribution			0.48
• ≤6	7 (25.0%)	132 (27.2%)	
• 7	14 (50.0%)	245 (50.4%)	
• ≥8	7 (25.0%)	109 (22.4%)	
Clinical Stage			0.55
• T1–T2a	17 (60.7%)	301 (61.9%)	
• T2b–T2c	8 (28.6%)	141 (29.0%)	
• T3–T4	3 (10.7%)	44 (9.1%)	
NCCN Risk Group			0.61
• Low	5 (17.9%)	105 (21.6%)	
• Intermediate	16 (57.1%)	275 (56.6%)	
• High	7 (25.0%)	106 (21.8%)	

MRI-only Pretraining Cohort: *n* = 312 ProstateX, PI-CAI; not genomically matched, used exclusively for encoder pretraining; Bullet points indicate subgroup distributions within clinical categories where applicable; * *p*-values reported for descriptive comparison only; Genomics+MRI. The cohort is underpowered for inferential testing.

**Table 8:** Low-Risk Control versus Higher-Risk/csPCa Discrimination Performance Across Settings. Genomics+WSI (*n* = 486): primary analysis, 5-fold CV with Bonferroni-corrected DeLong test. Genomics+MRI (*n* = 28): exploratory analysis, bootstrap CI reported; no inferential statistics. * *p* < 0.001 vs. all baselines (WSI setting only).

Model	AUROC (Genomics + MRI)	AUROC (Genomics + WSI)	F1 Score (WSI)	Sensitivity (WSI)	Specificity (WSI)	AUROC (Genomics Only)
MRI/WSI Only	0.945 ± 0.008	0.950 ± 0.009	0.932 ± 0.012	0.91 ± 0.03	0.93 ± 0.02	N/A
G Only	0.935 ± 0.010	0.935 ± 0.010	0.916 ± 0.014	0.89 ± 0.03	0.92 ± 0.03	0.935 ± 0.010
Early Fusion	0.960 ± 0.008	0.958 ± 0.009	0.943 ± 0.011	0.93 ± 0.02	0.94 ± 0.02	0.920 ± 0.012
Cross Attention	0.965 ± 0.007	0.962 ± 0.008	0.948 ± 0.010	0.94 ± 0.02	0.94 ± 0.02	0.930 ± 0.011
State of the Art	0.966 ± 0.007	0.963 ± 0.008	0.949 ± 0.010	0.94 ± 0.02	0.94 ± 0.02	0.928 ± 0.012
Ours	0.980 ± 0.006 ^†^	0.985 ± 0.005 *	0.972 ± 0.006	0.95 ± 0.02	0.96 ± 0.02	0.945 ± 0.010 *

^†^ indicates the second-best performance among all compared methods; * indicates the best performance in each column; N/A: not applicable, as this configuration does not include genomic input required for this evaluation setting.

**Table 9:** Risk-Stratification Performance (5-fold CV).

Metric	Genomics + MRI Setting	Genomics + WSI Setting	Degradation (Genomics-Only)
Weighted Accuracy	91.3% ± 1.2%	92.1% ± 1.1%	8.7%
Cohen’s κ	0.85 ± 0.03	0.86 ± 0.03	0.15 ± 0.05
Sensitivity (High Risk)	0.93 ± 0.02	0.94 ± 0.02	0.12 ± 0.05
Specificity (Low Risk)	0.92 ± 0.02	0.93 ± 0.02	0.10 ± 0.04
Severe Error Rate	4.1% ± 0.8%	3.8% ± 0.7%	+2.9%

**Table 10:** Clinical Significance Prediction Performance.

Calibration Metric	Genomics + MRI Setting	Genomics + WSI Setting	Baseline Best
Brier Score	0.095 ± 0.012	0.088 ± 0.011	0.135 ± 0.018
Expected Calibration Error	0.024 ± 0.004	0.021 ± 0.003	0.048 ± 0.007
Maximum Calibration Error	0.032 ± 0.005	0.028 ± 0.004	0.068 ± 0.009
NCCN Guideline Compliance	96.8% ± 1.2%	97.2% ± 1.1%	89.3% ± 2.3%

**Table 11:** Comprehensive Ablation Study Results from 5-fold CV.

Ablation Condition	Low-Risk vs. csPCa ΔAUROC	Risk-Stratification ΔAccuracy	Clinical Significance ΔBrier
No Contrastive	−0.041 ± 0.008 *	−3.2% ± 0.7%	+0.024 ± 0.006
No Clinical Constraints	−0.008 ± 0.003	−6.8% ± 1.1% *	+0.038 ± 0.008 *
Early Fusion Instead	−0.025 ± 0.006 *	−3.8% ± 0.8%	+0.015 ± 0.004
No Modality Dropout	−0.015 ± 0.004	−1.5% ± 0.6%	+0.009 ± 0.003
3D CNN (MRI)/ResNet (WSI)	−0.017 ± 0.005	−2.1% ± 0.6%	+0.013 ± 0.004
No Genomics Conditioning	−0.032 ± 0.007 *	−5.2% ± 0.9% *	+0.029 ± 0.007 *

* indicates statistically significant degradation (*p* < 0.05) compared to the full model baseline.

**Table 12:** Consistency Metrics Results.

Consistency Metric	Value	95% CI
Low-risk vs. csPCa Agreement	98.1%	[95.3%, 99.6%]
Risk-Stratification Agreement	94.3%	[90.8%, 96.9%]
Clinical Significance Agreement	96.8%	[93.7%, 98.7%]
Embedding Similarity	0.92 ± 0.03	[0.89, 0.95]
Modality Leakage	0.53 ± 0.03	[0.50, 0.56]

**Table 13:** XAI Validation Metrics.

Validation Metric	MRI Setting	WSI Setting	Expert Agreement
Lesion Overlap (Dice)	85.2% ± 4.3%	88.1% ± 3.9%	>85% target
Attention-AUC	0.92 ± 0.03	0.94 ± 0.03	>0.90 target
Clinical Relevance Score	4.5/5.0 ± 0.2	4.6/5.0 ± 0.2	>4.5/5.0 target
Inter-rater Reliability	0.87 ± 0.04	0.89 ± 0.03	>0.85 target

**Table 14:** Significant Genotype–Phenotype Correlations.

Genomic Alteration	MRI Correlation	WSI Correlation	Clinical Implication
PTEN Loss	r = 0.72 (*p* < 0.001)	Attention = 0.78 ± 0.06	Anterior aggression
ERG Fusion	r = 0.58 (*p* < 0.005)	Attention = 0.72 ± 0.07	Homogeneous growth
SPOP Mutation	r = 0.45 (*p* < 0.05)	Attention = 0.58 ± 0.08	Favorable prognosis
TP53 Mutation	r = 0.62 (*p* < 0.01)	Attention = 0.65 ± 0.06	Treatment resistance

**Table 15:** Computational Performance Metrics.

Metric	Genomics+MRI Setting	Genomics+WSI Setting	Improvement vs. Baselines
Training Time	35 ± 3 h	30 ± 2 h	28% faster
Inference Latency	1.9 ± 0.3 s	3.3 ± 0.4 s	Comparable
Memory Footprint	8.3 GB	7.9 GB	20% reduction
Parameters	42.7 M	42.7 M	33% fewer
Energy Consumption	3.4 kWh	2.9 kWh	24% reduction

**Table 16:** Multi-Task Learning Synergy Metrics.

Synergy Metric	Value	Interpretation
Parameter Efficiency Gain	27%	Reduced redundancy
Task Attention Correlation	0.75 ± 0.05	Strong task interdependence
Representation Overlap	71% ± 6%	Shared feature utilization
Convergence Acceleration	21% ± 4%	Faster training
Regularization Benefit	0.15 ± 0.03	Improved generalization

**Table 17:** Comparative Analysis of AI Frameworks for Prostate Cancer.

Ref	Task	Model	Data	Output	Key Results	MT	Int.	Main Limitation
[44]	Gland segmentation	Dual-path Swin-UNet	PANDA, SICAPv2	Segmentation	mDice: 0.95 (PANDA), 0.66 (SICAPv2)	No	No	Large cross-dataset performance gap
[45]	Multi-cancer classification	RL-SARSA (OCWLS)	Multi-omics	Classification	F1: 0.96 (carcinomas)	Yes	No	Not prostate-specific
[46]	Secure multi-cancer detection	FL + AE + XGBoost	Multi-omics	Classification	Acc: 98%, −61% delay	Yes	No	Focus on system metrics, not clinical
[47]	csPCa MRI segmentation	UNETR, Swin-UNETR	Multi-center MRI	Segmentation	Best voxel AUC: 0.77 ± 0.04	No	Yes	Robustness gains are limited at the voxel level
[48]	PCa vs. BPH diagnosis	ML ensemble	Serum, Urine proteomics	Classification	AUC: 0.87–0.88	No	Yes	Small cohorts
[49]	Pathological staging	RF, XGB, DL	TCGA-PRAD (RNA-seq)	Classification	RF F1 ≈ 83%	No	Partial	Multi-class staging difficulty
[50]	MRI grade classification	RMANet	ProstateX + local MRI	Classification	AUC: 0.84	No	Yes	The dataset is outdated
[51]	Artifact-robust MRI PCa	DNN + TPAS	Multi-center MRI	Classification	AUC: 0.87 (patient)	No	No	Targets only rectal artifacts
[52]	MRI AI benchmarking	Systematic review	12 open MRI datasets	Mixed	Acc up to 96.6%	—	Review	High reporting heterogeneity
[40]	Pathology foundation	CONCH	Diverse WSI datasets	Embeddings, classification	WSI-level AUROC up to 0.97 on TCGA subtyping	No	Yes	Not directly evaluated on our tasks
[41]	Pathology foundation	UNI	Massive WSI collection	Embeddings, classification	Strong zero-shot and fine-tuned performance on multiple cancer types	No	Yes	Not evaluated in multimodal setting
[42]	Multimodal survival	MCAT	TCGA (genomic + WSI)	Risk scores	c-index up to 0.73 across cancer cohorts	Yes	Yes	Survival endpoint, not classification
[43]	Missing-modality learning	MultiMAE	Various public datasets	Classification	Robustness gains under modality dropout	Yes	No	Not prostate-specific
**Ours (Genomics** **+MRI)**	Low-risk control vs. higher-risk/csPCa discrimination, Gleason risk stratification, csPCa prediction	Genomics-anchored multimodal DL (contrastive + clinical constraints)	TCGA-PRAD multi-omics + TCIA MRI (+ ProstateX/PI-CAI pretraining)	Multi-task classification	AUROC: 0.980 ± 0.006	Yes	Yes	Limited matched genomics–MRI cohort (*n* = 28)
**Ours (Genomics** **+WSI)**	Low-risk control vs. higher-risk/csPCa discrimination, Gleason risk stratification, csPCa prediction	Genomics-anchored multimodal DL (contrastive + clinical constraints)	TCGA-PRAD multi-omics + H&E WSI (*n* = 486)	Multi-task classification	AUROC: 0.985 ± 0.005	Yes	Yes	Retrospective validation; prospective study pending

(MT: Multi-task, Int.: Interpretability, AE: Autoencoder, FL: Federated Learning, and csPCa: Clinically significant prostate cancer).

## Data Availability

The datasets analyzed in this study are publicly available. Genomic data (RNA sequencing, copy-number variation, and DNA methylation) along with corresponding hematoxylin and eosin (H&E) whole-slide images were obtained from The Cancer Genome Atlas Prostate Adenocarcinoma (TCGA-PRAD) cohort via the Genomic Data Commons (https://portal.gdc.cancer.gov/, (accessed on 22 October 2025). Multiparametric MRI data were retrieved from The Cancer Imaging Archive (TCIA) under the TCGA-PRAD imaging collection (https://www.cancerimagingarchive.net/collection/tcga-prad/, (accessed on 22 October 2025). Additional prostate MRI datasets used for auxiliary pretraining, including ProstateX and PI-CAI, were obtained from TCIA and their respective publicly available challenge repositories under their original data access conditions. All datasets are de-identified and publicly available for research purposes. Processed data splits, model configurations, and implementation details will be publicly released upon publication to ensure reproducibility of the reported results.

## Abbreviations

The following abbreviations are used in this manuscript:

AI	Artificial intelligence
AUROC	Area under the receiver operating characteristic curve
csPCa	Clinically significant prostate cancer
ECE	Expected calibration error
mpMRI	Multiparametric magnetic resonance imaging
NCCN	National Comprehensive Cancer Network
PCa	Prostate cancer
PI-RADS	Prostate Imaging-Reporting and Data System
PSA	Prostate-specific antigen
TCGA	The Cancer Genome Atlas
TCGA-PRAD	The Cancer Genome Atlas Prostate Adenocarcinoma
TCIA	The Cancer Imaging Archive
WSI	Whole-slide image
